# Conventional Cementless Total Hip Arthroplasty for Hip Osteoarthritis after Distal Trochanteric Transfer: A Report of Two Cases

**DOI:** 10.1155/2020/8853453

**Published:** 2020-08-10

**Authors:** Hirohito Hirata, Motoki Sonohata, Akira Hashimoto, Sakumo Kii, Takema Nakashima, Masaya Ueno, Shuichi Eto, Shunsuke Kawano, Masaaki Mawatari

**Affiliations:** Department of Orthopaedic Surgery, Faculty of Medicine, Saga University, Saga 849-8501, Japan

## Abstract

Distal trochanteric transfer (DTT) has been widely performed to treat developmental dysplasia of the hip or Perthes disease. Total hip arthroplasty (THA) following DTT in patients with hip osteoarthritis is one of the most challenging procedures for hip surgeons, because great care must be taken regarding anatomical abnormalities of the greater trochanter and the soft tissue attached to the greater trochanter. To the best of our knowledge, there are no reports on THA after DTT. We herein report two cases of patients who underwent THA post DTT using cementless components. After THA, both patients developed abduction temporary contraction because of leg length extension and gluteus medius hypertension. However, in both cases, the contraction was reversible within two months and the final clinical result was good. Therefore, THA can be considered an effective and safe choice for treating osteoarthritis after DTT.

## 1. Introduction

Deformities of the proximal femur such as coxa vara and high-standing greater trochanter are known to occur because of conditions such as developmental dysplasia of the hip and Perthes disease [1]. Leg length discrepancy (LLD) and shortening of the abductor musculature can cause a reduction in the abductor lever arm and Trendelenburg gait [2–6]. Distal trochanteric transfer (DTT) has been widely performed to increase tension of the gluteal muscles and hip abductors [7, 8]. Although good results are reported with the procedure, studies have also shown that DTT does not prevent or lower the risk of the development of osteoarthritis of the hip [6, 9, 10]. In a previous study, 40% of patients who underwent DTT subsequently required hip arthroplasty for osteoarthritis [6]. THA for patients with hip osteoarthritis following osteotomy of the proximal femur, including DTT, is one of the most challenging procedures for hip surgeons [11, 12]. In particular, surgeons must take great care regarding anatomical abnormalities of the greater trochanter and soft tissue attached to the greater trochanter. However, there have been no reports regarding a focus on performing total hip arthroplasty (THA) after DTT. Therefore, it is unclear if conventional THA can yield satisfactory results in such a situation. Here, we report the results of conventional cementless THA after DTT.

This study protocol adhered to the ethical guidelines of the 1975 Declaration of Helsinki. The study was approved by our institutional review board. Both patients were informed that their cases would be submitted for publication, and they provided informed consent for the same.

## 2. Case Presentation

### 2.1. Case 1

A 56-year-old woman (height 156 cm, weight 46 kg) presented to our hospital with right hip pain, limited range of motion, and limping. Trendelenburg gait was observed. Her medical history included treatment for developmental dysplasia of the hip (DDH) with a cast when she was an infant. She developed hip joint pain and underwent DTT at the age of 23 years. Her hip pain improved after the surgery; however, she started to feel pain again when she was 40 years old.

**Figure 1:**
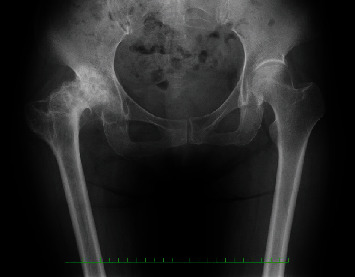
Radiographs of Case 1 before total hip arthroplasty.

Radiographs showed severe osteoarthritis of her right hip joint and the greater trochanter, which had moved to the level of the lesser trochanter (Figure 1). On anteroposterior radiography, a line was drawn through the teardrops and vertical from the teardrops. The vertical distance of the proximal lessor trochanter tip to this reference line was measured. The LLD was 36 mm.

**Figure 2:**
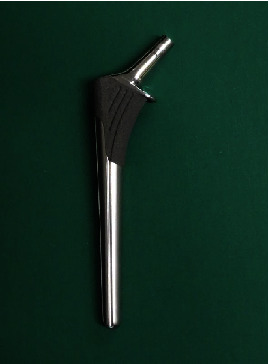
PerFix-HA femoral component used for implantation.

Conventional cementless THA was performed through a posterolateral approach with the patient in the lateral position under spinal anesthesia; the femoral component (PerFix-HA collared STD stem, neck-shaft angle 135°, Kyocera, Kyoto, Japan) (Figure 2) was used with a zirconia ball and an AMS-HA acetabular shell with a cross-linked ultra-high-molecular-weight polyethylene elevated liner (Kyocera, Kyoto, Japan).

Intraoperatively, as scar tissue augmentation was present due to the previous surgery, the surgeon paid close attention to identify the gluteus medius and minimus muscles and prevent damage to both muscles due to hypertension during the reduction after implant placement. The total operation time was 59 min, and the total blood loss was 675 g (intra and postoperative blood loss). The leg lengthening achieved was 18 mm, and 18 mm of the LLD persisted.

**Figure 3:**
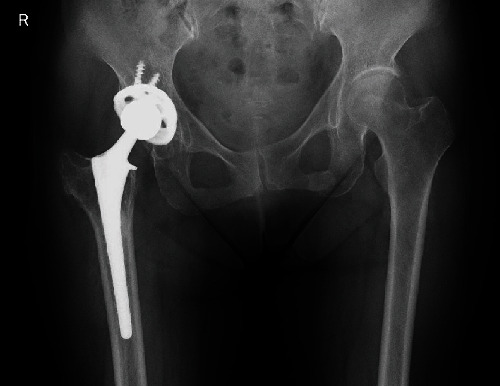
Radiographs of Case 1 last follow-up (7 years) after total hip arthroplasty.

The patient was allowed to stand and walk with full weight bearing after drain removal, 2 days after the operation. The pain improved remarkably; however, she presented with LLD due to an abduction contraction following extension of the leg length. However, the abduction contraction improved within two months under physical therapy. No other complications were observed, including dislocation, neurovascular abnormality, or infection. Final radiographs at 7 years after THA did not show subsidence of the stem or any type of loosening around the cup or stem (Figure 3).

We used the Japanese Orthopedic Association (JOA) hip score to evaluate the hip joint [13]. The JOA hip score includes four categories that amount to a total of 100 points as follows: pain (40 points), range of motion (20 points), walking (20 points), and activities of daily living (20 points). The preoperative JOA hip score was 46 points, which improved to 96 points at 7 years after surgery. Trendelenburg gait had improved.

### 2.2. Case 2

A 50-year-old woman (height 155 cm, weight 60 kg) first presented to our hospital with left hip pain, limited range of motion, and limping. Trendelenburg gait was observed. She did not have a history of treatment for DDH. She developed hip joint pain and underwent right DTT at the age of 20 years. Her hip pain had improved after the surgery; however, she started to feel pain again at 2 years after DDT. She underwent right-sided THA at another hospital at the age of 47 years.

**Figure 4:**
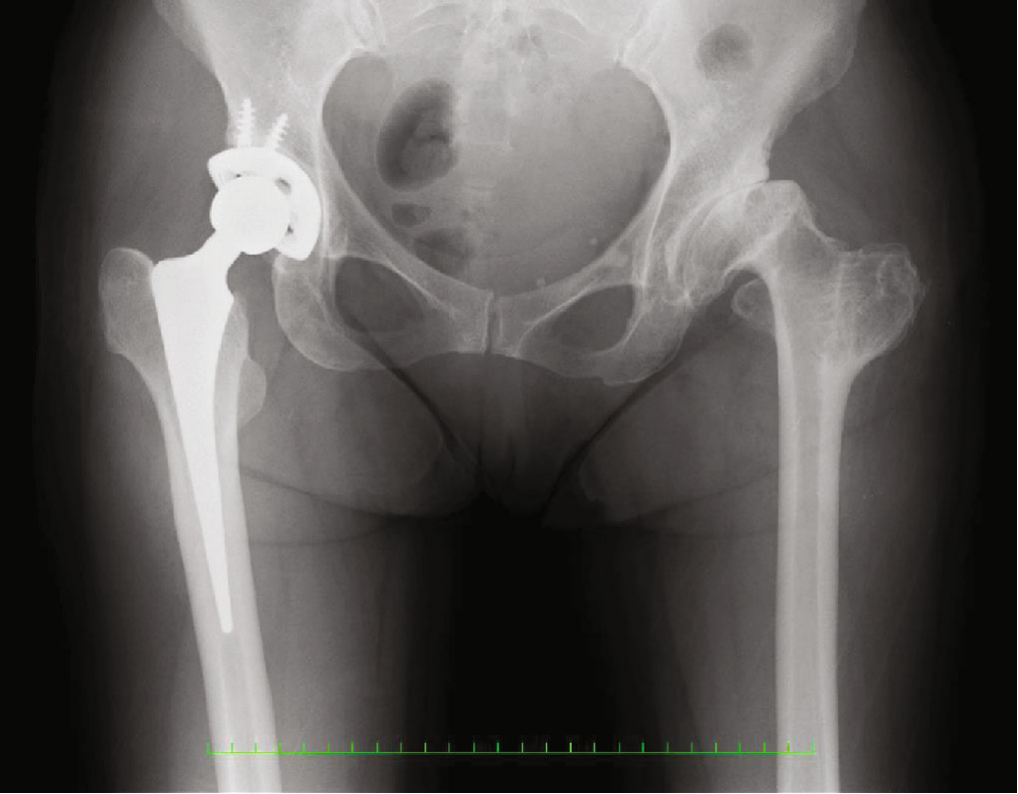
Radiographs of Case 2 before left total hip arthroplasty.

Radiographs showed severe osteoarthritis of her left hip joint and the left greater trochanter, which had moved to the level of the lesser trochanter. The past THA on the right hip joint was visible as well (Figure 4). The LLD was 55 mm.

The right THA was performed at the age of 47, and the left THA was performed at the age of 50. The THA procedures comprised conventional cementless THA in the same way as in Case 1 (anesthesia, surgical approach, implants, and rehabilitation).

Intraoperatively, the surgeon had to pay close attention to identify the gluteus medius and minimus muscles and prevent damage to the same muscle due to hypertension during the reduction after implant placement, similar to the circumstances of case 1. The operation time was 60 min, and the total blood loss was 488 g (intra and postoperative blood loss). The leg lengthening was 27 mm, and 28 mm of the LLD persisted.

**Figure 5:**
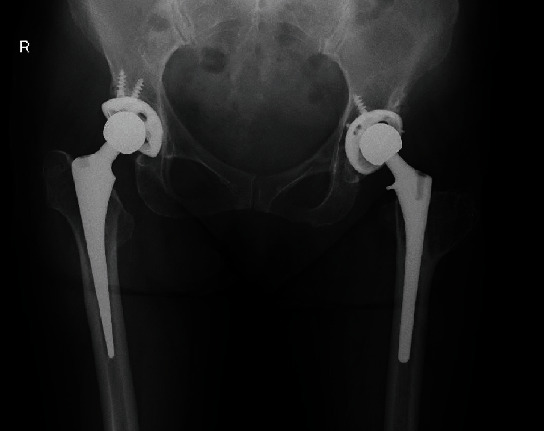
Radiographs of Case 2 at their last follow-up (11 years) after total hip arthroplasty.

As in Case 1, the pain improved remarkably; however, the patient presented with LLD due to an abduction contraction following extension of the leg length. The abduction contraction improved within two months under physical therapy. No other complications, such as dislocation, neurovascular abnormality, or infection, were observed. Final radiographs at 11 years after left THA did not show subsidence of the stem or any type of loosening around the cup or stem (Figure 5). The preoperative JOA hip score was 39 points, which improved to 71 points at 11 years after surgery. Trendelenburg gait had improved.

## 3. Discussion

Both the cases showed good results of THA following DTT. However, postoperative temporary abduction contracture was caused by the leg extension in both cases. DTT is known to be associated with an increase in hip abductor tension [11, 12]; unfortunately, this often means that DTT needs additional THA [6, 9, 10]. It is well-known that THA following osteotomy of the proximal femur involves many technical difficulties as compared to primary THA [11, 12]. There are some reports on THA for hip osteoarthritis following proximal femoral valgus osteotomy, proximal femoral varus osteotomy, Schanz osteotomy, and anterior rotational osteotomy of the femoral head [11, 12, 14, 15]; however, few reports exist on THA following DTT.

Surgeons should be aware of the following 2 surgical pitfalls. First, maximum attention should be paid when exposing the joint capsule to avoid muscle damage; the gluteus medius and minimus muscles are present distal to the joint capsule and are therefore vulnerable to damage. As both the gluteus medius and minimus muscles may have been damaged by previous surgery, manipulation should be done with caution. Damage to these muscles can lead to formation of a limp after THA.

Second, stem selection is an extremely important part of the procedure. The conventional cementless femoral component we used in both surgeries has been classified as a fit and fill type and is designed for the purpose of increasing the intramedullary occupancy rate of the femur and increasing the initial fixation [16]. Since the greater trochanter is positioned distally, it does not have the shape of the normal intertrochanteric crest; therefore, securing stability by using the proximal metaphyseal fit type component is considered difficult.

In both cases, postoperative abduction contracture occurred and the patients developed LLD. In addition to extension of the leg length, it is thought that this is due to the decrease in soft tissue flexibility, including that of the gluteus medius muscle, due to the scar tissue that is often formed around the gluteus medius following DTT. Fortunately, abduction contracture in both patients improved significantly within two months under physical therapy, and the final clinical results were excellent.

Although both patients were allowed to stand and walk with full weight bearing from the early postoperative period, the final radiographs did not show subsidence or loosening. Therefore, it seems that the stem used in both cases can be considered appropriate for THA following DTT.

We herein described two cases of THA following DTT. Although THA was needed to have more attention during operations, it has become clear that it is a safe and effective treatment for hip osteoarthritis following DTT. Furthermore, it was suggested that postoperative abductor contracture was more likely than usual THA, but the abductor contracture was reversible.
